# Non-invasive MRI of brain clearance pathways using multiple echo time arterial spin labelling: an aquaporin-4 study

**DOI:** 10.1016/j.neuroimage.2018.12.026

**Published:** 2019-03

**Authors:** Yolanda Ohene, Ian F. Harrison, Payam Nahavandi, Ozama Ismail, Eleanor V. Bird, Ole P. Ottersen, Erlend A. Nagelhus, David L. Thomas, Mark F. Lythgoe, Jack A. Wells

**Affiliations:** a UCL Centre for Advanced Biomedical Imaging, Division of Medicine, UCL, London, UK; b GliaLab and Letten Centre, Division of Physiology, Department of Molecular Medicine, Institute of Basic Medical Sciences, University of Oslo, Oslo, Norway; c Neuroradiological Academic Unit, UCL Institute of Neurology, UCL, London, UK; d Leonard Wolfson Experimental Neurology Centre, UCL Institute of Neurology, UCL, London, UK

**Keywords:** Aquaporin-4, Multiple echo-time, ASL, Blood-brain interface, Blood-brain barrier, Water permeability, Glymphatic system

## Abstract

There is currently a lack of non-invasive tools to assess water transport in healthy and pathological brain tissue. Aquaporin-4 (AQP4) water channels are central to many water transport mechanisms, and emerging evidence also suggests that AQP4 plays a key role in amyloid-β (Aβ) clearance, possibly via the glymphatic system. Here, we present the first non-invasive technique sensitive to AQP4 channels polarised at the blood-brain interface (BBI). We apply a multiple echo time (multi-TE) arterial spin labelling (ASL) MRI technique to the mouse brain to assess BBI water permeability via calculation of the exchange time ($T_{ex}^{w}),\;$ the time for magnetically labelled intravascular water to exchange across the BBI. We observed a 31% increase in exchange time in AQP4-deficient (*Aqp4*^*−/−*^) mice (452 ± 90 ms) compared to their wild-type counterparts (343 ± 91 ms) (p = 0.01), demonstrating the sensitivity of the technique to the lack of AQP4 water channels. More established, quantitative MRI parameters: arterial transit time (δ_a_), cerebral blood flow (CBF) and apparent diffusion coefficient (ADC) detected no significant changes with the removal of AQP4. This clinically relevant tool may be crucial to better understand the role of AQP4 in water transport across the BBI, as well as clearance of proteins in neurodegenerative conditions such as Alzheimer's disease.

## Graphical abstract

**Figure:**
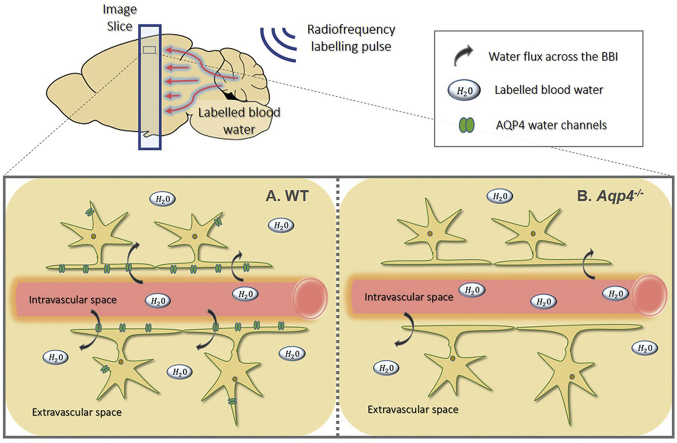

## 1 Introduction

Regulating brain water transport is vital to brain homeostasis and dysfunction is associated with several neurological conditions such as meningitis, traumatic brain injury and cerebral oedema (Papadopoulos and Verkman, 2005; Manley et al., 2000, 2004; Chen et al., 2016). Parenchymal osmoregulation is supported by brain aquaporin-4 (AQP4) channels, trans-membrane proteins that are highly polarised to astrocytic endfeet, that facilitate bi-directional flux of water (Nagelhus and Ottersen, 2013; Papadopoulos and Verkman, 2013). A brain-wide waste removal pathway, coined the glymphatic system, describes the exchange of cerebrospinal fluid (CSF) and interstitial fluid (ISF), a transition strongly mediated by AQP4. AQP4 channels are critical to glymphatic function and appear to play a key role in clearance of deleterious proteins such as amyloid-β (Aβ) (Iliff et al., 2012). Studies report ∼70% decrease in glymphatic function in mice lacking AQP4 channels, which likely contributes to the measured ∼55% decrease in rates of Aβ clearance from the brain parenchyma in the same AQP4-deficient animals (Iliff et al., 2012). Further studies indicate that when AQP4 no longer remains polarised to the astrocytic endfeet there is a reduction in glymphatic function (Kress et al., 2014). In agreement, loss of perivascular AQP4 is associated with accumulation and misagregation of amyloid (Zeppenfeld et al., 2017; Yang et al., 2011). Many of the methods for assessing the role of AQP4 in brain clearance pathways are highly invasive and not clinically applicable (Papadopoulos and Verkman, 2005; Iliff et al., 2012; Haj-Yasein et al., 2011); therefore the development of non-invasive tools for the assessment of AQP4 expression/polarisation would be highly beneficial to advance our understanding of AQP4 mediated clearance of waste products and neurotoxic proteins from the brain. Ultimately this may lead to new approaches for early diagnosis and effective therapeutic intervention in neurodegenerative diseases.

To address the lack of non-invasive tools available to assess water transport mechanisms, we have built on our previous work in the rat brain and have developed a multiple echo time (multi-TE) arterial spin labelling (ASL) MRI technique (Wells et al., 2016). Multi-TE ASL is able to estimate rates of labelled vascular water delivery across the blood-brain interface (BBI) in the mouse brain parenchyma, as a surrogate index of BBI permeability to water. ASL techniques use endogenous blood water as a tracer (by magnetically labelling arterial blood water as it flows into the brain), conventionally to map rates of brain tissue perfusion (Detre et al., 1992; Williams et al., 1992; Koretsky, 2012). However, multi-TE ASL uses the difference in transverse relaxation times (T2) between the intravascular (IV) compartment (T2_IV_ ∼20  ms at 9.4T) (Lee et al., 1999; Vazquez et al., 2010) and extravascular (EV) static tissue (T2_EV_ ∼37  ms at 9.4T) (Kara et al., 2013), to enable dynamic assessment of labelled blood water crossing the BBI. The time for magnetically labelled blood water to exchange from the IV compartment to EV brain tissue is quantified via the ‘exchange time’ parameter ($T_{ex}^{w})$ (Wells et al., 2013), a quantitative surrogate measure of BBI permeability to water. Altered BBI water permeability may provide an early indication of neuropathology (Kim et al., 2008) and therefore has potential as a clinically viable biomarker of diseases such as Alzheimer's Disease.

While there are several transport mechanisms for water passing across the BBI (MacAulay and Zeuthen, 2010; Tait et al., 2008), AQP4 provides a ‘path of least resistance’ which supports the rapid flux of water molecules into the brain parenchyma (Manley et al., 2000; Haj-Yasein et al., 2011; Amiry-Moghaddam et al., 2003). The absence of AQP4 has a marked effect on BBI permeability to water, as reported by using invasive techniques (Papadopoulos and Verkman, 2005). An important motivation for this novel methodological development was the potential to apply this technique to genetic mouse models of neurodegeneration and other models of specific BBI abnormality. In this study, we have developed multi-TE ASL for the mouse brain and applied the technique to *Aqp4*^*−/−*^ mice to estimate BBI permeability to water. The genetic removal of AQP4 channels allows controlled experimental conditions to assess the sensitivity of the technique to record AQP4-mediated water flux across the BBI. We hypothesize that impaired water transport mechanisms in *Aqp4*^*−/−*^ mice will be detectable via an associated increase in water exchange time. If so, the non-invasive multi-TE ASL technique may provide a novel tool to assess AQP4 polarisation at the BBI in order to better understand the dynamic role of AQP4 in water transport mechanisms and brain clearance pathways.

## 2 Method

### 2.1 Experimental set-up

All experiments were performed in mice in accordance with the European Commission Directive 86/609/EEC (European Convention for the Protection of Vertebrate Animals used for Experimental and Other Scientific Purposes) and the United Kingdom Home Office (Scientific Procedures) Act (1986). All mice were acclimatised two weeks prior to data acquisition in an animal house maintained at a temperature of 21 ± 2 °C and a relative humidity of 55 ± 10%, on a 12 h light/12 h dark cycle with food and water provided *ad libitum*. Female C57/BL6 WT mice (Charles River Laboratories) at 18 ± 3 weeks were used to establish the techniques for non-invasive assessment of BBI permeability to water in the mouse brain (n = 5 at TI = 400 ms and n = 6 for all other inflow times (note, at TI = 1500 ms the data acquisition was only possible in five mice due to practical reasons)). Male *Aqp4*^*−/−*^ mice at 6 months old (n = 9) (Thrane et al., 2011) and male C57/BL6 WT age-matched controls (n = 9) (Charles River Laboratories) were used to evaluate the contribution of AQP4 in BBI permeability to water.

All animals were induced in 2% isoflurane anaesthetic in a mixture of 1.0 L/min medical air, and the isoflurane level was manually adjusted throughout the scans to maintain the respiration rate at ∼100 bpm. Core body temperature was monitored using a rectal probe (SA Instruments) and maintained at 37 ± 0.5 °C via regulation of an adjustable temperature water bath. Animals were secured into the MRI cradle with a nose cone, ear bars and bite bar to minimise head movement during the scan.

### 2.2 Image acquisition

Images were acquired using a 9.4T VNMRS horizontal bore MRI scanner (Agilent Inc.) with an Agilent 205/120HD gradient set. A 72 mm inner diameter volume coil and a two channel array surface coil (Rapid Biomedical) were used for RF transmission and reception respectively. Prior to data acquisition, 1st and 2nd order shimming was performed to reduce the magnetic field inhomogeneities across the brain. An anatomical reference scan was acquired using a T2-weighted multi-slice Fast Spin Echo sequence with parameters: 30 slices, slice thickness = 0.6 mm, field-of-view = 35 × 35 mm, matrix size = 256 × 256, effective echo time (TE) = 48 ms, repetition time (TR) = 4s, echo train length = 8. The main ASL imaging protocol was based on a flow-alternating inversion recovery (FAIR) sequence, with global inversion width = 216 mm, slice-selective width = 16 mm with a two-shot segmented spin-echo echo planar imaging (SE-EPI) readout. Further sequence parameter details are provided below.

Analysis was performed using Matlab R2015a (Mathworks Inc.). Mean ASL images were generated by a pairwise subtraction of the control and labelled images at each echo time or inflow time, and averaged across all repetitions.

#### 2.2.1 Arterial transit time and cerebral blood flow estimation

The arterial transit time (δ_a_) reflects the arrival time of the labelled bolus of blood water to the imaging region of interest and in this work δ_a_ was estimated using a separate multiple short inflow time (multi-TI) ASL acquisition. At short inflow times the ASL signal, ΔM, fits to a linear model against TI, according to the pulsed arterial spin labelling (PASL) biophysical model (Buxton et al., 1998):

(1) ΔM=0whenδa>TI

(2) $$\Delta M\left(TI\right)=A.\left(TI\;-\;\text{δa}\right)\;.exp\left(-\frac{TI}{T1_{a}}\right)\;\;\;\text{when}\;\text{δa}\;<\;\text{TI}$$

where A is a constant factor, dependent on CBF (A = 2α. M_OA_. CBF/λ), T1_a_ is the longitudinal relaxation of the arterial blood and M_0A_ is the equilibrium magnetisation of the blood. A direct fit of $\Delta M\left(TI\right).\text{exp}\left(TI/T1_{a}\right)\;$ as a function of TI therefore enables simultaneous estimation of both $\delta_{a}$ and CBF.

The multi-TI protocol used the following sequence parameters: TI = 200 ms, 300 ms, 400 ms, 500 ms; echo time (TE) = 12 ms; repetition time (TR) = 5000 ms, field-of-view (FOV) = 25 × 25 mm; matrix size = 32 × 32; number of k-space line (k_0_) = 20, repetitions = 20, (repetitions = 10 for assessment of AQP4 contribution for BBI permeability).

#### 2.2.2 Multi-TE acquisition protocol for technique validation

A multi-TE ASL sequence was applied with imaging parameters: inflow times (TI) = 400 ms, 1000 ms, 1500 ms and 3500 ms, each acquired with a range of TE = 15, 23, 30, 40, 50, 65 ms; TR = 5000 ms except for TI = 3500 ms where TR = 7000 ms, FOV = 25 × 25 mm; matrix size = 32 × 32; k_0_ = 20, repetitions = 20.

#### 2.2.3 Single compartment model

Apparent transverse relaxation, T2_app_, was estimated by fitting the ASL signal decay as a function of echo time to a simple mono-exponential model, and reflects the relative contributions of the intravascular (IV) space and the extravascular (EV) tissue to the overall ASL signal (Lee et al., 1999).

For T2_app_ analysis, ASL signal was extracted from a manually defined cortical region of interest (ROI) was fitted to a monoexponential model to extract cortical T2_app_ for each subject, at each inflow time. T2_app_ maps were generated by fitting the ASL images across all echo times to a monoexponential decay for visualisation purposes.

#### 2.2.4 Two compartment model

The multi-TE ASL technique was extended to separate IV-EV compartments by fitting the ASL signal decay to a two compartment bi-exponential model at intermediate inflow times (1000 ms and 1500 ms).

(3) $$\Delta M\;=\;\Delta M_{IV}\;\exp\left(-\frac{TE}{T2_{IV}}\right)+\Delta M_{EV}\;\exp\left(-\frac{TE}{T2_{EV}}\right)$$

where ΔM_IV_ and ΔM_EV_ are intravascular and extravascular ASL signal weighting factors respectively, TE is the echo time, T2_IV_ is the T2 value of the IV arterial compartment and T2_EV_ is the T2 value of the EV tissue (Lee et al., 1999). T2_EV_ is determined from a monoexponential decay fit to the control image data from, under the assumption that the control images were dominated by EV effects (following the previous approach described in reference (Wells et al., 2013)).

The ASL signal weighting factors enable estimation of the intravascular fraction at a given inflow time:

(4) $$Intravascular\;fraction=\frac{\Delta M_{IV}}{\Delta M_{IV}+\;\Delta M_{EV}}$$

The kinetic perfusion model proposed by Alsop and Detre (1996) and adapted by Wang (Wang et al., 2002) was applied to ASL signal weighting factors to estimate the tissue transit time, δ. The adapted two compartment kinetic perfusion model (Wells et al., 2013; Buxton et al., 1998; Alsop and Detre, 1996) is described by the following equations:

(5) $$\Delta M_{IV}=\;\frac{2M_{0}f}{\lambda }\left\{exp\left(-TI\times R1_{a}\;\right)\;\left[\min\left(\left(\delta_{a}-TI+\tau ,\;0\right)\;–\;\delta_{a}\right)\;–\;(\text{min}\left(\delta -TI+\tau ,\;0\right))\;–\;\delta \right]\right\}$$

(6) $$\Delta M_{EV}=\;\frac{2M_{0}f}{\lambda }\left\{exp\left(-TI\times R1_{app}\;\right)\;\left[\frac{\exp\left(\text{min}\left(TI,\;\delta +\tau \right)\Delta R\right)\;-\exp\left(\delta \Delta R\right)}{\Delta R}\right]\right\}$$

where M_0_ is the equilibrium of the magnetisation, f is the CBF, λ is the blood-brain partition coefficient (λ = 0.9 (Herscovitch and Raichle, 1985)), TI is the inflow time, R1_a_ is the longitudinal relaxation rate derived from the arterial compartment, assumed here to be 1/2.4s (Dobre et al., 2007), R1_app_ is the longitudinal relaxation rate of the tissue, here fixed at R1_app_ = 1/1.7s (Wells et al., 2013), ΔR = R1_app_ – R1_a_, and τ is the temporal length of the tagged bolus τ = 2.0s inferred from previous experiments in our laboratory (Wells et al., 2015).

The exchange time (T_ex_^w^) indicates the time for magnetically labelled vascular water to exchange across the BBI into brain tissue once the labelled bolus reaches the imaging slice:

(7) $$T_{ex}^{w}=\;\delta -\;\delta_{a}$$

The exchange time parameter provides a quantitative, surrogate marker to BBI permeability to water. Table 1 and Table 2 found in the Appendix shows the measured variables and the assumed parameters respectively for the models used in the analysis.

**Fig. 1:**
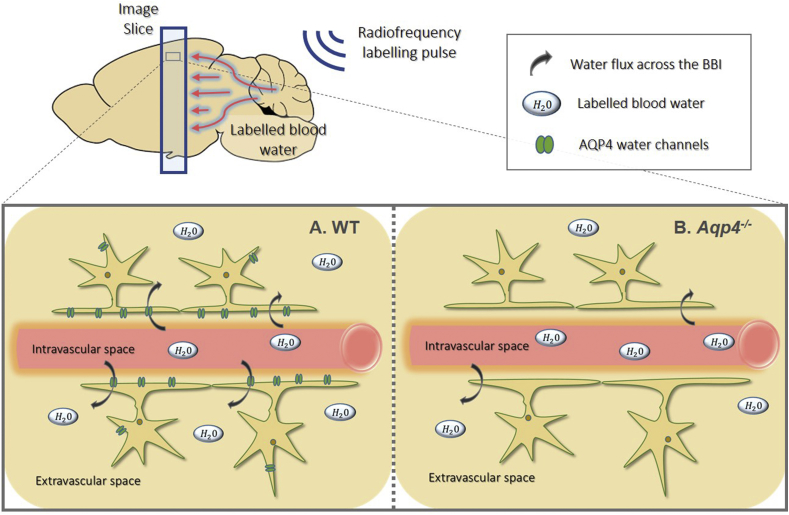
**Schematic of ASL labelling scheme and labelled blood water transfer from intravascular space to extravascular tissue space. A.** Labelled blood water molecules exchange into the extravascular space via all water transport mechanisms including aquaporin-4 (AQP4) water channels in wild-type (WT) mice. **B.** In the absence of AQP4, in AQP4-deficent mice (*Aqp4*^*−/−*^), the water transport mechanisms are restricted and only occur through cotransport with organic molecules and by diffusion through the lipid bilayer of the plasma membrane.

Multi-TE ASL was implemented to measure the water flux across the BBI to assess AQP4 contribution, Fig. 1 shows schematic of the technique applied to the WT and *Aqp4*^*−/−*^ mice. Imaging parameters: TE = 15, 18, 23, 30, 40, 50, 65 ms; TI = 1500 ms; FOV = 25 × 25 mm; matrix size = 32 × 32; slice thickness = 2 mm; TR = 5000 ms; partial Fourier acquisition (16 + 4 k-space line acquired); repetitions = 15.

**Fig. 2:**
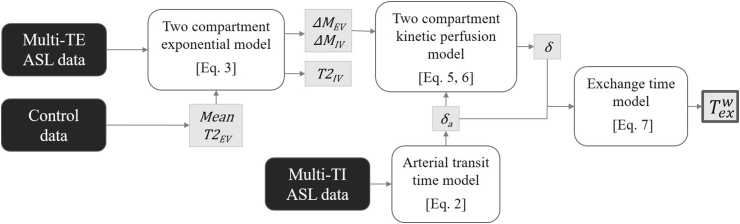
Flowchart for the analysis pipeline to determine exchange time ($T_{ex}^{w})$ from multi-TE ASL data, multi-TI ASL data and control data.

For $T_{ex}^{w}$ measurements, ASL signal intensity from the multi-TE ASL data and mean T2_EV_, were derived from the same cortical brain region, and all data sets were fit with two compartment exponential model (Eq (3)). The outputs of the fitting process were the individual ΔM_IV_ and ΔM_EV_ contributions for each subject and a single common T2_IV_ across all of the subjects. The individual ΔM_IV_ and ΔM_EV_ contributions were used to calculate the intravascular fraction, Eq (4). ASL signal from the short multi-TI data, extracted from the same manually defined cortical ROI, was fitted to the linear, arterial transit time model, Eq (2), where extrapolation through the inflow time axis yields δ_a_ for the individual animals. ΔM_IV_ and ΔM_EV_ signal weightings were used together with the independent measurement of δ_a_ to extract tissue transit time (δ) from the two compartment kinetic perfusion model, to be used to calculate the final cortical exchange time ($T_{ex}^{w}$). Fig. 2 shows flowchart of the multi-step analysis pipeline for $T_{ex}^{w}$ measurements. The initial exchange time measurements from the animals in the protocol validation group was calculated using two TI values (1000 ms and 1500 ms) and was averaged across both TIs to increase accuracy.

The whole brain region for each subject was manually segmented to generate a mean exchange time map for each animal group for visualisation. This was achieved by fitting the two compartment model using T2_EV_ maps, arterial transit time maps, and fixed mean cortical T2_IV_ for each individual animal. The ventricular regions were manually segmented and excluded from the analysis since the two compartment model fails within this region as there is no ASL signal. The exchange time maps were registered together and averaged for each animal group.

#### 2.2.5 Apparent diffusion coefficient

Apparent diffusion coefficient (ADC) estimates the molecular motion of water in the targeted brain region, and provides an indication of parenchymal water mobility. This was evaluated to assess any tissue differences in measured ADC of the parenchymal tissue between the *Aqp4*^*−/−*^ and WT animals. ADC was estimated using a two b-value approach with diffusion gradients applied in a single slice select direction: b-values = 0 and 1030.5 s/mm^2^; TE = 23.82 ms; TR = 2500 ms; data matrix = 64 × 64; slice thickness = 1.0 mm; repetitions = 10 for both b-values and spin-echo EPI readout.

Diffusion images were averaged across all of the repetitions for each b-value. The diffusion images were used to extract the signal from cortical ROI corresponding to the same region used to measure $T_{ex}^{w},$ δ_a_ and CBF. Diffusion-weighted signal was fitted to the standard mono-exponential diffusion model to estimate the ADC.

### 2.3 Quantification of Aqp4 Gene Expression

For quantification of *Aqp4* mRNA expression in cortical brain region, three WT mice and three *Aqp4*^*−/−*^ mice were euthanised by overdose with sodium pentobarbital (10 ml/kg, intra-peritoneal), the brain rapidly removed, hemisected, and the cortex dissected and snap frozen in isopentane pre-chilled on dry ice.

Total RNA from each brain region was extracted using the RNeasy^®^ Plus Microkit (Qiagen, UK). Total RNA was converted to cDNA using the QuantiTect^®^ Reverse Transcription Kit (Qiagen), which was quantified using the Eppendorf Mastercycler with Realplex software (v1.5, Eppendorf, UK) and the TaqMan^®^ Gene Expression master mix (Applied Biosystems, UK). TaqMan^®^ Gene Expression assays for *Aqp4* and reference housekeeper genes (*ACTB* and *GAPDH*) were used, and *Aqp4* expression level determined using the 2^–ΔΔCt^ method (Livak and Schmittgen, 2001), normalising to expression in the wild-type cortex.

All data are reported as the mean and associated error (± standard deviation). All analysis was performed using GraphPad Prism 6 (GraphPad Software). A Kruskal-Wallis test was performed on the T2_app_ data at different inflow times. A one-tailed paired students t-test was performed on the intravascular fractions measured at TI = 1000 ms and TI = 1500 ms. A one-tailed student's t-test was performed on data from WT and *Aqp4*^*−/−*^ animals to compare $T_{ex}^{w}$, and two-tailed students's t-test was performed on the data to compare δ_a_, CBF, ADC and mRNA expression measurements. For all tests, p < 0.05 was considered to indicate a statistically significant result.

## 3 Results

### 3.1 Multi-TE ASL for non-invasive assessment of BBI permeability to water in the mouse brain: acquisition at multiple inflow times

**Fig. 3:**
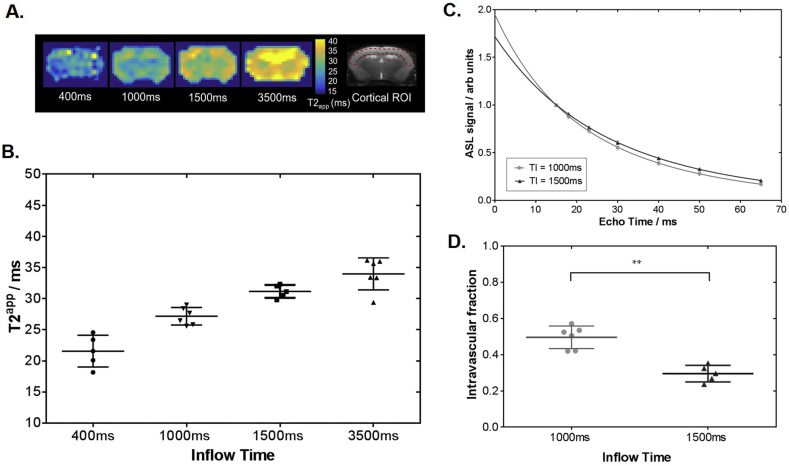
**Modelling the multi-TE ASL signal**. **A.** Representative T2_app_ map at increasing inflow times (indicated on each image) for a single subject, with an anatomical reference image shown with cortical ROI in red, for illustration purposes. **B.** T2_app_ at increasing inflow times fitted to single compartment model for individual subjects. **C.** The mean cortical signal across all subjects fitted to the two compartment model at TI = 1000 ms and 1500 ms. **D.** The intravascular fraction of the ASL signal determined for the individual subjects at TI = 1000 ms and 1500 ms using the two compartment model. The mean parameter and associated error (±std) are displayed with each marker representing the individual animals. *** indicates p < 0.001.

We observe a progressive increase in T2_app_ with inflow time in the cortical brain region from 21.6 ± 2.6  ms at TI = 400 ms to 34.0 ± 2.6  ms at TI = 3500 ms (p = 0.0003) (Fig. 3B), with representative T2_app_ maps at each inflow time (Fig. 3A). T2_app_ at TI = 400 ms corresponds to arterial blood T2 at 94.2% oxygen saturation from previous calibration experiments at 9.4T (Lee et al., 1999) and T2_app_ at TI = 3500 ms was 34.0 ± 2.6 ms, which tends closely towards the T2 value of cortical tissue (∼37 ms). This provides evidence that the technique is sensitive to the transfer of labelled blood water moving across the BBI into the extravascular tissue of the mouse brain as TI increases.

A relatively balanced contribution from both the intravascular (IV) and extravascular (EV) compartments was demonstrated at TI = 1000 ms and 1500 ms with T2_app_ = 27.2 ± 1.4 ms and T2_app_ = 31.2 ± 1.0 ms respectively, which lie between T2_IV_ ∼ 20 ms (Lee et al., 1999; Vazquez et al., 2010) and T2_EV_ ∼ 37 ms (Kara et al., 2013). Hence, the analysis was extended by fitting the ASL signal to a bi-exponential model to separate the IV and EV signal contributions at these intermediate TIs (Fig. 3C). The intravascular fraction confers the proportion of labelled blood water residing in the IV compartment with ‘fast-decaying’ signal derived from the arterial blood (T2_IV_). A significant reduction in the intravascular fraction from 0.50 ± 0.07 to 0.30 ± 0.06 is reported as the TI increases from 1000 ms to 1500 ms (p = 0.0005) (Fig. 3D). We note consistency between the T2_IV_ of 16.0 ± 1.7  ms at 1000 ms and to 16.3 ± 2.6  ms at 1500 ms. The mean arterial transit time (δ_a_) was calculated to be δ_a_ = 157 ± 29 ms (see Supplementary Figure 1A), and from the fit to the kinetic two compartment perfusion model, mean cortical exchange time was calculated to be 395 ± 54 ms.

### 3.2 Application of Multi-TE ASL to evaluate contribution of AQP4 in BBI permeability to water

**Fig. 4:**
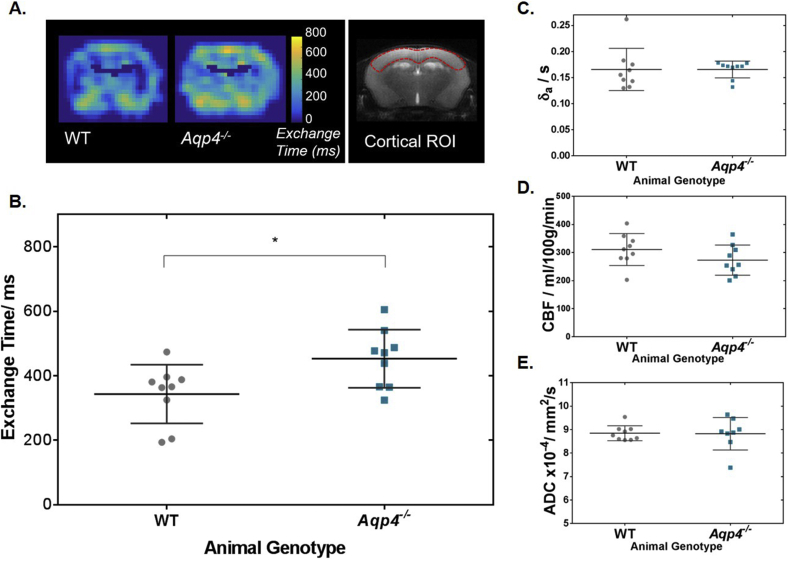
Time of exchange of labelled blood water into cortical tissue. **A.** Average exchange time maps for all animals (scale bar shown) and an anatomical reference of the cortical brain region for individual analysis, for illustration purposes. **B.** The mean cortical exchange time measurements for individual animals. **C.** Mean cortical arterial transit time (δ_a_) for individual animals. **D.** Mean cortical CBF for individual animals **E.** Mean cortical ADC measured in individual animals. Group mean parameter and associated error (±std) are displayed (n = 9). * indicates p < 0.05.

Using the multi-TE ASL imaging protocol to map the exchange times, we observed a 31% increase in $T_{ex}^{w}$ in cortical brain tissue of the AQP4-deficient mice when compared to the WT mice (452 ± 90 ms and 343 ± 91 ms respectively; p = 0.01) (Fig. 4A and B). This reflects slower movement of labelled vascular water into the extravascular cortical tissue in the absence of AQP4 channels. Expression of AQP4 was confirmed using quantitative real time polymerase chain reaction (qRT-PCR) (Supplementary Figure 2). T2_IV_ measured at 19.7 ± 3.3 ms in WT animals and 20.4 ± 2.3 ms in *Aqp4*^*−/−*^ animals. T2_EV_ from the control data measured at 35.8 ± 1.3 ms in WT animals and T2_EV_ = 37.4 ± 0.8 ms in *Aqp4*^*−/−*^ animals.

There is no detectable difference in the arterial transit time of vascular water to the cortical brain region between WT mice, δ_a_ = 166 ± 40 ms and *Aqp4*^*−/−*^ mice, δ_a_ = 166 ± 16 ms (p = 0.535) (Fig. 4C). This provides evidence that there are no marked differences in the arterial vascular architecture in the two animal cohorts. The arterial transit times are also consistent with those previously measured in the separate cohort of WT mice (Supplementary Figure 1). Mean cortical CBF was 310 ± 57 ml/100  g/min in WT mice and 273 ± 54 ml/100  g/min in *Aqp4*^*−/−*^ mice (p = 0.188), which also suggests there to be preserved cerebral haemodynamic between both animal group (Fig. 4D). Taken together this indicates that the measured differences in the cortical exchange time between animal groups are not due to alterations in the dynamics of the arterial blood supply. There are no measurable differences in parenchymal water mobility between the two groups as ADC values from the same cortical brain region were 8.85 ± 0.32 × 10^−4^ mm^2^/s in WT mice and 8.83 × 10^−4^ mm^2^/s in *Aqp4*^−/−^ mice (p = 0.869) (Fig. 4E). The data shows that the absence of AQP4 water channels has no significant impact on δ_a_, CBF or ADC measurements, which is consistent with previous reports indicating a relatively mild phenotype of this mouse model which, with some exception (Nagelhus and Ottersen, 2013), only displays clear differences to WT animals in pathophysiological conditions (Papadopoulos and Verkman, 2013; Amiry-Moghaddam and Ottersen, 2003).

## 4 Discussion

In this study, we have developed a non-invasive technique to assess blood-brain interface (BBI) permeability to water in the mouse brain, using multiple echo time (multi-TE) ASL. Our results suggest that the technique is sensitive to the presence of AQP4, as a 31% reduction of the estimated rate of water flux across the BBI was detected in the absence of these water channels. We recorded no significant changes in other, more established, quantitative MRI parameters: arterial transit time (δ_a_), cerebral blood flow (CBF) and apparent diffusion coefficient (ADC) with the lack of AQP4 water channels. This highlights the sensitivity of the multi-TE ASL approach in the non-invasive assessment of AQP4 mediated water permeability in the brain. The emerging roles that AQP4 plays in brain clearance pathways point to AQP4 as a novel imaging target for neurological conditions such as Alzheimer's Disease.

Water traverses several different interfaces within the brain, for example: across the blood-brain barrier (BBB) and the astrocyte foot processes into extravascular brain tissue, through ependymal layer to access the ventricular space from the interstitial space and via the subpial astrocyte processes and pial cells into the subarachnoid space. All three routes are thought to be important for maintaining the water homeostasis of the brain and AQP4 is highly expressed in each of these interfaces (Vindedal et al., 2016). AQP4 channels are believed to be a key component of the glymphatic clearance pathway, a waste solute removal system dependent on glial water transport (Nedergaard, 2013). It has been shown that glymphatic function was impaired by ∼70% in *Aqp4*^*−/−*^ mice compared to the WT animals, as detected by movement of a fluorescent tracer (Iliff et al., 2012). In this study we demonstrate sensitivity of multi-TE ASL to AQP4 expression, which plays a critical role in the glymphatic pathway. As such, this technique may represent a surrogate marker of glymphatic function. Further understanding the contributions of the rate of water flux across different membrane interfaces could help to detect putative malfunctions of these pathways in disease conditions.

The current study represents the first demonstration of a non-invasive technique able to detect differences in rates of water flux across the BBI due to the differential expression of AQP4. An overview of the literature suggests that prominent phenotypes associated with the AQP4-model mainly emerge in pathological conditions, such as induced meningitis or ischemic stroke (Papadopoulos and Verkman, 2005, 2013; Manley et al., 2000; Nagelhus and Ottersen, 2013). A marked decrease in brain water uptake was observed in *Aqp4*^*−/−*^ mice, measured ex-vivo by the change in brain water content, as an index of BBI water permeability (Papadopoulos and Verkman, 2005). The changes observed here were not as pronounced. However, the present recordings were done non-invasively and without subjecting the animals to pathophysiological conditions.

While *Apq4*^*−/−*^ mice appear to have a subtle phenotype, they show a slight increase in brain water content (Haj-Yasein et al., 2011), which may account for the increase in T2_EV_ in the cortical brain region of this animal group. Further, *Aqp4*^*−/−*^ animals have an intact endothelium as shown by histology (Saadoun et al., 2009), and confirmed by the absence of leakage of Evans Blue dye into the brain parenchyma (Haj-Yasein et al., 2011). This highlights the sensitivity of the multi-TE ASL technique in the ability to detect differences in BBI water flux without disruption to the integrity of the endothelial wall. It has been reported that small interference RNA (siRNA) can be used to transiently knock down AQP4 expression in the rat brain which caused a significant decrease in the measured ADC (Badaut et al., 2011). Interestingly, in the present study we recorded no significant changes in the ADC of the tissue in the *Aqp4*^*−/−*^ mouse. This difference may reflect the acute nature of AQP4 modulation using siRNA, compared to the chronic AQP4-deficient model examined here. Overall, the specific contribution and possible compensation of other water transport mechanisms on water mobility are not fully understood.

The underlying mechanisms that determine changes to BBB permeability to water remain poorly understood. Diffusion-weighted ASL techniques (DW-ASL) have been used to assess BBB water permeability in various neurological conditions such as stroke (Tiwari et al., 2016), sleep apnea (Palomares et al., 2015) and brain tumours (Wang et al., 2006, 2007). An increase in BBB water permeability via water exchange rate (K_w_), was shown in a rat model of ischemic stroke, where changes in blood water permeability covered more extensive regions compared to the changes in BBB permeability determined by Evans Blue (Tiwari et al., 2016), demonstrating that water may be a more sensitive marker for BBB permeability than larger exogenous tracers. A further study reported a significant reduction of K_w_ in patients with obstructive sleep apnea (OSA) (Xie et al., 2013). In this study the authors speculated that the measured reduction in water exchange rate reflects an increase in BBB permeability to water; however it is possible that compromised aquaporin function associated with this condition caused the measured reduction in the permeability-surface area product to water (PS_w_). This highlights the need to better understand the role of AQP4 in water flux across the BBI, measured using translational non-invasive imaging techniques, which is the aim of the current work.

Changing the chosen inflow time (TI) provides a means of modulating the proportion of labelled water molecules in the intravascular (IV) and extravascular (EV) compartments during the image acquisition phase. We hypothesised that as we increased the TI, the proportion of labelled water in the EV compartment would increase, as greater time is allowed for the labelled bolus of IV water to exchange across the BBI. Consistent with this hypothesis, we observed a gradual increase in T2_app_ as TI was increased from 400 to 3500 ms (see Fig. 3B). These measurements provide evidence that the technique presented here is sensitive to the movement of labelled blood water across the BBI into extravascular tissue. The results in this study are comparable to previous T2_app_ data from rat cortical brain tissue (Wells et al., 2013), but measurements reported here have been extended to both shorter and longer inflow times to further isolate the likely source of the labelled blood water to the IV or EV compartment respectively.

The two compartment model is successful at separating the IV and EV compartments when a relatively blanced contribution of labelled blood water resides within each compartment. At intermediate inflow times of TI = 1000 ms and TI = 1500 ms, the proportion of labelled blood water in the IV compartment measured by the intravascular fraction are 0.5 and 0.3 respectively. The intravascular fraction is comparable to the relative amount of labelled water reported in the human brain, where intravascular fraction was measured at 0.39, 0.26 and 0.15 at TI = 800 ms, 1200 ms and 1500 ms respectively, when ASL was combined with a diffusion-weighted (DW) MRI sequence (Wang et al., 2006).

The estimates of the IV fraction at TI = 1000 ms and 1500 ms were integrated into a kinetic model to calculate the tissue transit time (δ) for each mouse. In order to account for possible differences in arrival time, in this study, the arterial transit time (δ_a_) was measured in a separate, short TI, acquisition. The mean cortical exchnage time, $T_{ex}^{w}$, was measured at 395 ± 54 ms, providing a quantitative, surrogate index of BBI permeability to water. The $T_{ex}^{w}$ measurement is highly similar to that of the rat cortical brain tissue (Wells et al., 2013), estimated using a similar approach. $T_{ex}^{w}$ is also comparable to the transfer time parameter (T_bl-ex_) derived from the grey matter in the human brain where T_bl-ex_ was 440 ± 30 ms, using a T2-ASL method (Gregori et al., 2013). Further work must be done to relate these values to establish a gold standard measurement. This could be achieved using radiolabelled water with positron emission tomography (PET), although it is unknown whether such techniques are able to distinguish changes to such rapid water exchange timescales as observed here (i.e mean exchange time = 343 ms (WT) vs 452 ms (*Aqp4*^*−/−*^)). However, to our knowledge, this is the first report of the non-invasive assessment of BBI permeability to water in the mouse brain which provides opportunity for future application to models of neurodegenerative disease and specific BBI abnormalities.

Multi-TE ASL is attractive because it offers a non-invasive approach to assess BBI water permeability. Conventionally, BBB permeability dysfunction is measured using dynamic contrast enhanced (DCE) MRI which requires contrast agents such as gadolinium. DCE-MRI detects gross BBB defects when a marked breakdown of endothelial tight junctions permits large contrast agent molecules to egress into the brain parenchyma. Detecting changes in BBI permeability to water may identify distinct pathological processes at the BBI (e.g. loss of AQP4 polarisation) that may occur at earlier stages of neurodegenerative disease progression prior to the opening of the endothelial layer. DW-ASL techniques have been used to determine the water exchange rate, K_w_ (Tiwari et al., 2016; St. Lawrence et al., 2012), reported at 252 ± 38min^−1^ (Tiwari et al., 2016) in rat brain, 193 ± 50 min^−1^ (Wang et al., 2006) and 110 ± 18 min^−1^ (St. Lawrence et al., 2012) in human grey matter and 126 ± 18 min^−1^ (St. Lawrence et al., 2012) in human white matter. Preliminary comparisons between the exchange time and K_w_ metrics, using an adapted single pass approximation model (St. Lawrence et al., 2012; St Lawrence et al., 2000) applied to mouse cortical brain tissue show reasonable similarity (K_w_ = 141 ± 22 min^−1^) to the other species. Multi-TE ASL and DW-ASL eliminates the need to inject MRI tracers for permeability measures, and though these agents are used routinely in the clinical setting, there is increasing speculation about the potential harm that repeated doses may cause to patients in the long term (Gulani et al.). Therefore, there may be further clinical benefits to non-invasive measure of BBI permeability.

Adapted ASL methods that use alternate contrast mechanisms to assess BBB permeability to water have also been proposed in various studies (St. Lawrence et al., 2012; Zhou et al., 2001; Liu et al., 2011; Ewing et al., 2001). DW-ASL techniques may be complicated by the directional dependence of the signal attenuation, which is dependent on the chosen inflow time (Wells et al., 2016). A recent study estimated the water extraction fraction (E) by measuring the proportion of labelled spins draining to large cerebral veins for BBB water permeability determination (Lin et al., 2018). This approach provides limited spatial specificity and may restrict the regions where BBB permeability can be assessed, because of the necessity to extract the signal from the large draining veins. Visual inspection of Fig. 4A indicates spatial heterogeneity of the exchange time within the coronal slice studied in this work. The present study was designed to measure water exchange time in the cortical region and the modulation of this parameter by AQP4 expression. Future studies, however, may wish to further probe the regional distribution of water exchange time, possibly by incorporating a multi-slice approach to image acquisition. Further experiments would be necessary to explore how other factors such as distribution of tight junction proteins, pericyte coverage or extent of AQP4 polarisation impact the water flux across the BBI.

There is increasing evidence to suggest that AQP4 water channels plays an important role in brain Aβ clearance (Iliff et al., 2012; Xu et al., 2015). The loss of perivascular localization of AQP4 is proposed to be a factor that is associated with an increased Aβ burden (Zeppenfeld et al., 2017; Yang et al., 2011). Studies show a correlation between AQP4 polarisation with the severity of AD in the human brain (Zeppenfeld et al., 2017).The data suggests that the multi-TE ASL technique applied here is sensitive to the perivascular expression of AQP4, given that the technique is specifically targeting water exchange at the BBI. Future studies applying multi-TE ASL to other genetic mouse models could more specifically address the effect of specific AQP4 pools on the transfer of water across the BBI.

In conclusion, we have developed a multi-TE ASL technique for the mouse brain to assess AQP4-mediated water transport at the BBI. Previous studies suggest that the capacity of this water transport system is a key determinant of Aβ clearance from the brain. The emerging importance of AQP4-mediated clearance pathways, such as the glymphatic system, make this technique a promising and clinically applicable tool for better understanding the role of AQP4-mediated clearance in pathological conditions like Alzheimer's Disease.

## Funding

This work is supported by the Medical Research Council (MR/K501268/1), the EPSRC-funded UCL Centre for Doctoral Training in Medical Imaging (EP/L016478/1) and the UCL Leonard Wolfson Experimental Neurology Centre (PR/YLR/18575). JW is supported by the Wellcome Trust/Royal Society Sir Henry Dale Fellowship (204624/Z/16/Z).

## Figures and Tables

**Table 1:** Parameters measured from the data.

Measured variables	
Exchange time	$T_{ex}^{w}$
Tissue transit time	δ
Arterial transit time	$\delta_{a}$
Cerebral blood flow	f
Intravascular ASL signal	$\Delta M_{IV}$
Extravascular ASL signal	$\Delta M_{EV}$
Intravascular transverse relaxation	$T2_{IV}$
Extravascular transverse relaxation	$T2_{EV}$
Apparent transverse relaxation	$T2_{\text{app}}$
Apparent diffusion relaxation	ADC

**Table 2:** Assumed variables used in the models.

Assumed variables		Assumed Value
Blood-brain partition coefficient	λ	0.9
Longitudinal relaxation rate (blood)	${\text{R}1}_{\text{a}}$	1/2.4s
Longitudinal relaxation rate (tissue)	${\text{R}1}_{\text{app}}$	1/1.7s
Temporal length of tagged bolus	τ	2.0s

## Footnotes

Appendix Supplementary data related to this article can be found at https://doi.org/10.1016/j.neuroimage.2018.12.026.
