# Supplementary material for: Non-invasive MRI of brain clearance pathways using multiple echo time arterial spin labelling: an aquaporin-4 study
Source: Neuroimage. 2019 Mar;188:515–23. doi: 10.1016/j.neuroimage.2018.12.026 (PMC6414399; doi:10.1016/j.neuroimage.2018.12.026)
Supplement: Multimedia component 1 [file mmc1.docx]

## Supplementary Figures

### Arterial transit time and Cerebral Blood Flow


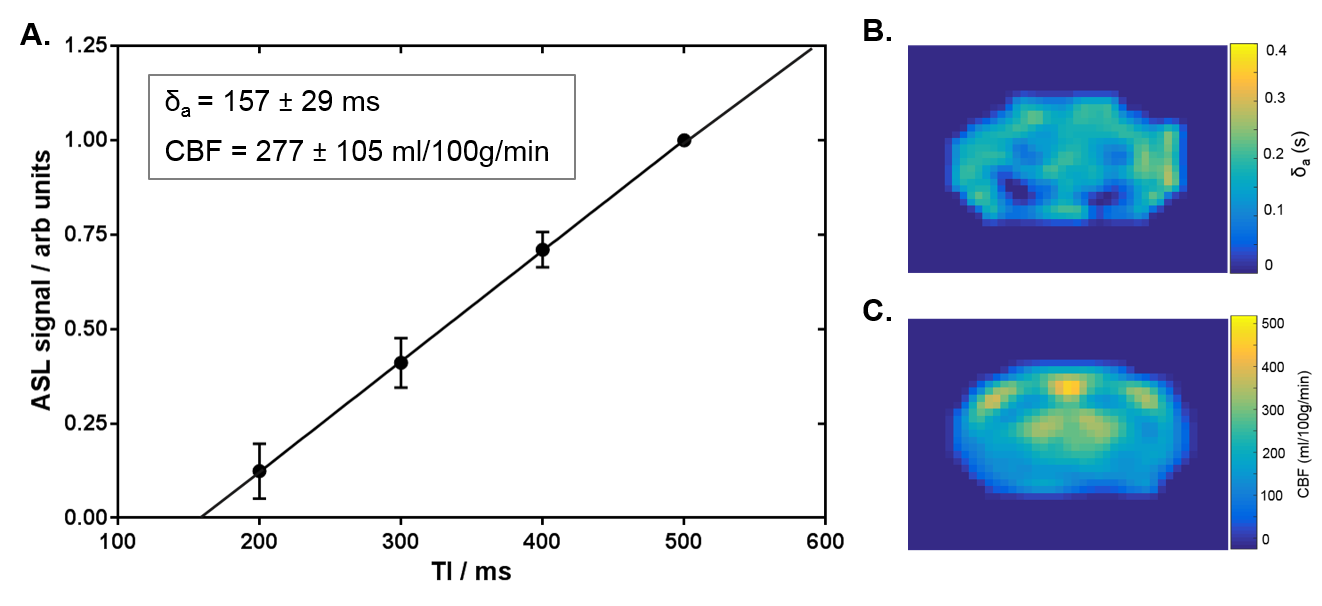


***Supplementary Figure 1: Modelling ASL signal at short inflow times. A.*** *The normalised mean cortical signal across all subjects fitted to a linear model with mean value and associated error (± std) displayed.* ***B.*** *Representative arterial transit time (δ_a_) map for individual subject.* ***C.*** *Representative CBF map for individual subject. Respective scale bars are displayed.*


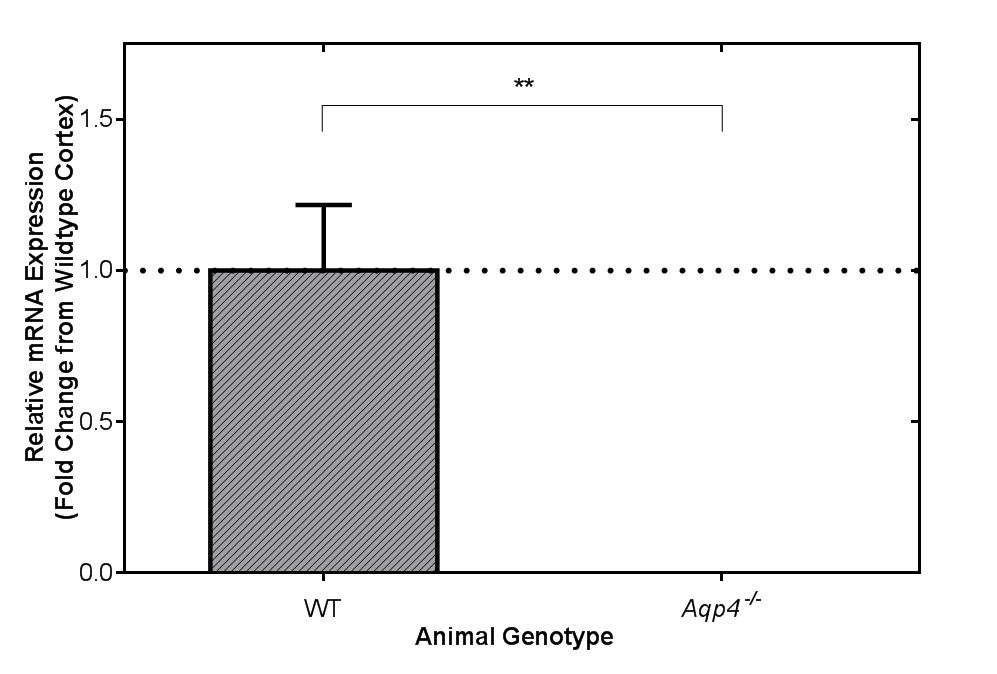


***Supplementary Figure 2: Aqp4 mRNA expression in the cortex of wildtype (WT) and Aqp4^-/-^mice***
